# Drug-induced sedation endoscopy (DISE) classification systems: a systematic review and meta-analysis

**DOI:** 10.1007/s11325-017-1521-6

**Published:** 2017-06-06

**Authors:** Esuabom Dijemeni, Gabriele D’Amone, Israel Gbati

**Affiliations:** 1 Research and Development Department, DISE INNOVATION, 24 Park Central Building, Bow Quarters, 60 Fairfield Road, London, UK; 2 Department of Bioengineering, Imperial College London, London, UK; 3 Dyson School of Design Engineering, Imperial College London, London, UK; 4 School of Design, Royal College of Art, London, UK; 5 Department of Mechanical Engineering, Imperial College London, London, UK

**Keywords:** Drug-induced sedation endoscopy, Classification, Sleep apnea, Review, Upper airway obstruction, Sleep

## Abstract

**Purpose:**

Drug-induced sedation endoscopy (DISE) classification systems have been used to assess anatomical findings on upper airway obstruction, and decide and plan surgical treatments and act as a predictor for surgical treatment outcome for obstructive sleep apnoea management. The first objective is to identify if there is a universally accepted DISE grading and classification system for analysing DISE findings. The second objective is to identify if there is one DISE grading and classification treatment planning framework for deciding appropriate surgical treatment for obstructive sleep apnoea (OSA). The third objective is to identify if there is one DISE grading and classification treatment outcome framework for determining the likelihood of success for a given OSA surgical intervention.

**Methods:**

A systematic review was performed to identify new and significantly modified DISE classification systems: concept, advantages and disadvantages.

**Results:**

Fourteen studies proposing a new DISE classification system and three studies proposing a significantly modified DISE classification were identified. None of the studies were based on randomised control trials.

**Conclusion:**

DISE is an objective method for visualising upper airway obstruction. The classification and assessment of clinical findings based on DISE is highly subjective due to the increasing number of DISE classification systems. Hence, this creates a growing divergence in surgical treatment planning and treatment outcome. Further research on a universally accepted objective DISE assessment is critically needed.

## Introduction

Locating the level of upper airway obstruction is a key clinical feature for assessing obstructive sleep apnoea [1]. Obstructive sleep apnoea (OSA) is the most prevalent sleep disorder breathing problem. The first description of the clinical features of obstructive sleep apnoea was in 1976 [2]. OSA is characterised by repetitive partial or complete obstruction of the upper airway during sleep. This results in the reduction or cessation of airflow. This may lead to repetitive hypoxia, increased retention of carbon dioxide and arousals to restore upper airway patency. Hence, sleep is fragmented. One definition of an apnoea is a total obstruction of the airway where there is a complete blockage for 10 s or more. A hypopnea is a partial obstruction of the airway where there is a partial blockage with airflow reduction of greater than 50% for 10 s or more.

A comprehensive sleep history is vital prior to embarking on investigations to confirm the diagnosis of OSA. The information gained from a sleep history is related to intoxications, medications, weight management, family history, medical history, surgical history, sleep behaviour, and nocturnal and daily symptoms. The Epworth sleepiness scale (ESS) can be used to assess the severity of daytime sleepiness [3]. However, there is a weak correlation between ESS and OSA severity [4]. The gold standard technique for diagnosing obstructive sleep apnoea is polysomnography (PSG) [5]. A full-night PSG is considered as the most accurate technique for measuring the presence and severity of OSA. During a PSG study, brain waves, muscle tone, chest and abdomen movement, airflow, heart rate, saturated blood oxygen level, sound level and sleep behaviour are monitored. PSG provides a robust physiological description of obstructive sleep apnoea. However, it fails to capture and assess anatomical information on the upper airway during an obstructive sleep apnoea. When surgical intervention on an obstructive upper airway is necessary, information provided by PSG is inadequate [6].

Drug-induced sedation endoscopy (DISE) also called sleep nasendoscopy (SNE) is a technique for direct visualisation of the site or sites of obstruction in sleeping patients [7]. DISE has shown that obstructive apnea occurs at mulitsegmental sections of the upper airway: nasopharynx, oropharynx and hypopharynx [8–10]. DISE has shown to be a quality assessment of dynamic upper airway event [11] and provides useful information on patient management [12], improved treatment planning [13], reliable intraobserver agreement [14], moderate to substantial interrater reliability [15] and good test-retest reliability [16]. Furthermore, DISE has been validated using bispectral monitoring [17] and showed good agreement with polysomnography [18].

Some criticism of DISE includes change in snoring pattern [19]; merely a ‘snapshot’ of snoring, not representative of how snoring changes throughout the sleep [20]; physiological sleep differs from sedation induced sleep; sedation leads to greater upper airway muscle relaxation; investigation is subjective; multiple classification systems and good analysis depends on experience [21]. However, DISE is currently the best technique for observing dynamic upper airway.

The null hypothesis for this study is that there is a universally accepted DISE classification system for analysing DISE findings. Ideally, this classification system should be able to accurately analyse DISE findings, provide a framework for surgical planning and predict the likelihood of OSA surgical treatment outcome. The alternative hypothesis for this study is that there are many DISE classification systems for analysing DISE findings. Hence, there is no objective and agreeable way of analysing DISE findings, diversity in surgical treatment planning and varying likelihood of OSA surgical treatment outcome.

The three key objectives for this system review and meta-analysis are as follows: to identify if there is a universally accepted DISE classification system for analysing DISE findings, to identify if there is one DISE classification treatment planning framework for deciding appropriate surgical treatment for OSA and to identify if there is one DISE classification treatment outcome framework for determining the likelihood of success for a given OSA surgical intervention. Further objective is as follows: identifying the data structure required and mode of analysis.

## Methods

The preferred reporting items for systematic reviews and meta-analysis (PRISMA) statement and checklist were followed as much as possible during this review [22].

### Study selection

The inclusion criteria for this review are the following: published studies proposing a new DISE classification system between 1991 and 2016 and published studies proposing a significant modification to an existing DISE classification system between 1991 and 2016. The exclusion criteria are the following: studies proposing an upper airway obstruction classification not based on DISE findings, DISE classification studies for children, further publications following an original publication of a DISE classification system and DISE studies that did not propose a new DISE classification system.

### Data collection process

Three authors (E.D., G.A. and I.G.) searched the international literature independently, identified the potentially relevant articles and used the inclusion and exclusion to determine which studies met criteria. Differences in opinion were resolved via consensus, and the plan was for the first author (E.D.) to make the final decision if there was a lack of consensus.

### Quality evaluation

The Oxford Centre for Evidence-based Medicine Level of Evidence was used to evaluate the quality of each study (Table 1) [23].

**Table 1:** Oxford Centre for Evidence-based Medicine Levels of Evidence

Level	Therapy
1a	Systematic review of randomised controlled trials
1b	Individual randomised controlled trial
2a	Systematic review of cohort studies
2b	Individual cohort study
2c	‘Outcomes research’
3a	Systematic review of case-control studies
3b	Individual case-control study
4	Case series (with or without comparison)
5	Expert opinion

## Results

### Study selection/characteristics

**Fig. 1:**
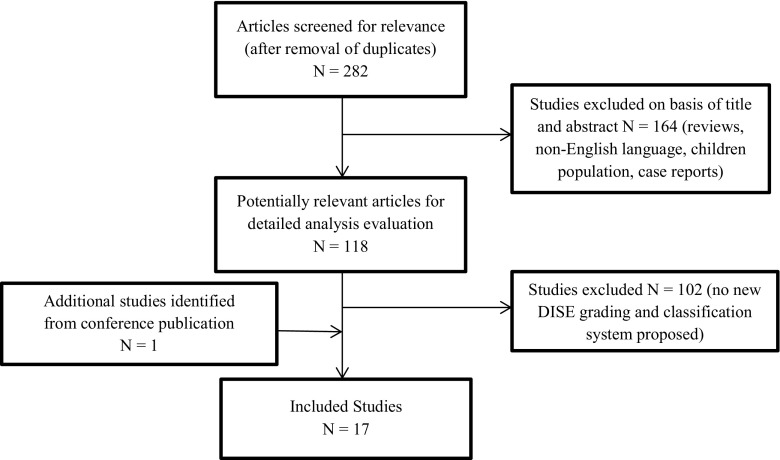
Flow diagram of study selection

Figure 1 illustrates the flow diagram of study selection. The systematic review of the literature provided a total of 282 potentially relevant studies. After screening the studies, 118 published studies were downloaded for full-text review. Seventeen studies met the pre-defined systematic review search criteria and were included in this meta-analysis. The main characteristics of these studies are summarised in Table 2. All the identified DISE classification systems are summarised in Table 3.

**Table 2:** Main characteristics of the selected studies

Study	Year	Number	Design	Study site	Level of evidence	Semi-quantitative/qualitative	Information type for classifier	Numerical score framework	Surgical treatment plan framework		Surgical outcome indicator	Data set provided	Method of analysis
Croft [7]	1991	71	Case series	UK	4	Qualitative	Anatomical only	No	No		No	No	Human
Pringle [24]	1993	90	Case series	UK	4	Qualitative	Anatomical only	No	Yes		Yes	No	Human
Camilleri [25]	1995	52	Case series	UK	4	Qualitative	Anatomical only	No	Yes		Yes	No	Human
Quinn [26]	1995	54	Case series	UK	4	Qualitative	Anatomical only	No	No		No	No	Human
Sadaoka [9]	1996	50	Case series	Japan	4	Qualitative	Anatomical only	No	No		No	No	Human
Higami [27]	2002	21	Case series	Japan	4	Qualitative	Anatomical only	No	Yes		Yes	No	Human
Iwanaga [28]	2003	60	Case series	Japan	4	Qualitative	Anatomical only	No	Yes		Yes	No	Human
Kezirian [29]	2011	–	Case series	USA, Germany, Netherlands	4	Qualitative	Anatomical only	No	No		No	No	Human
Vicini [30]	2012	–	Case series	Italy	4	Semi-quantitative	Anatomical only	Yes	No		No	No	Human
Bachar [32]	2012	55	Case series	Israel	4	Qualitative	Anatomical only	Yes	No		No	No	Human
Victores [33]	2012	24	Case series	USA	4	Qualitative	Anatomical only	No	No		Yes	No	Human
Gillespie [34]	2013	38	Case series	USA	4	Qualitative	Anatomical only	No	Yes		No	No	Human
Koo [35]	2013	69	Case series	Korea	4	Qualitative	Anatomical only	No	No		No	No	Human
Lee [36]	2015	85	Case series	Korea	4	Semi-quantitative	Anatomical only	No	No		No	No	Human
Herzog [37]	2015	40	Case series	Germany	4	Semi-quantitative	Anatomical only	No	No		No	No	Human
Carrasco-Llatas [14]	2016	31	Case series	Spain	4	Qualitative	Anatomical only	No	No		No	No	Human
Veer [38]	2016	–	case series	UK	4	Semi-quantitative	Anatomical only	Yes	No		No	No	Human

**Table 3:** Summary of DISE classification system

Author, year	Classification					
Croft, 1991 [7]	Group		Description			
	Group 1		Simple palatal snoring			
	Group 2		Single-level palatal obstruction			
	Group3		Multisegment obstruction			
Pringle, 1993 [24]	Grade		Description			
	Grade 1		Simple palatal snoring			
	Grade 2		Single-level palatal obstruction			
	Grade 3		Multisegmental involvement–intermittent oro-hypopharyngeal obstruction			
	Grade 4		Sustained multisegmental obstruction			
	Grade 5		Tongue base-level obstruction			
Camilleri, 1995 [25]	Grade		Description			
	Grade 1		Palatal snoring			
	Grade 2		Mixed snoring			
	Grade 3		Non-palatal (tongue base) snoring			
Quinn, 1995 [26]	Zone		Configuration			
	Palate		Funnel-shaped aperture			
			Transverse slit cross-sectional reduction			
	Tonsil		Hypertrophic tonsil medicalization			
	Tongue		Vibration against the pharyngeal wall			
	Epiglottis		Omega-shaped collapse			
			Fall back onto and vibrate against the posterior pharyngeal wall			
Sadaoka, 1996 [9]	Group		Description			
	Group A		Single-level palatal obstruction			
	Group B		Single-level tongue-based obstruction			
	Group C		Multisegment involvement			
Higami, 2002 [27]	Type		Description			
	Falling type		Soft palate and tongue sank due to gravity			
	All-round type		Submucosa of the pharynx very thick; hypertrophied tonsils covered in thick mucosa			
	Bilateral type		Hypertrophy of the palatine tonsils; enlarged tonsil buried in the arch of the palate			
Iwanaga, 2003 [28]	Obstruction type		Description			
	Soft palate		Uvula and soft palate come into contact with the posterior pharyngeal wall			
	Circumferential palatal		Full-circumference soft palatal airway obstruction involving the posterior and lateral pharyngeal walls			
	Tonsillar		Palatine tonsils come into contact at the midline during expiration			
	Root of tongue		Single-level obstruction at the root of tongue			
	Mixed		Soft palatal obstruction and tongue-based obstruction			
Kezirian, 2011 [29]	Structure	Degree of obstruction	Configuration			
	Velum	0, no obstruction; 1, partial obstruction; 2, complete obstruction	Anteroposterior	Lateral	Concentric	
	Oropharynx lateral walls + tonsils		X, not visualised	X, not visualised	X, not visualised	
	Tongue base		Anteroposterior	Lateral	X, not visualised	
	Epiglottis		Anteroposterior	Lateral	X, not visualised	
Vicini, 2012 [30]	Zone	Grade	Configuration			
	Nose	0–25%: 1 25–50%: 2 50–75%: 3 75–100%: 4	–	–	–	
	Oropharynx		Anteroposterior	Lateral	Concentric	
	Hypopharynx		Anteroposterior	Lateral	Concentric	
	Larynx (supraglottis, glottic)	Positive or negative obstruction	–	–	–	
Bachar, 2012 [32]	Zone	No collapse	Partial collapse	Complete collapse		
	Nose + nasopharynx	0	1	2		
	Palate (tonsils included)	0	1	2		
	Tongue base	0	1	2		
	Larynx	0	1	2		
	Hypopharynx	0	1	2		
Victores, 2012 [33]	Level	Degree		Sustainability		
		Partial	Complete	Intermittent	Sustained	
	Palate (velum)	p	P	–	–	
	Lateral wall/tonsillar pillars	l	L	–	–	
	Tongue base	t	T	1	2	
	Epiglottis	e	E	1	2	
Gillespie, 2013 [34]	Grade					
		0	1	2	3	4
	DISE index					
	Palate AP	No collapse	Partial collapse	Complete collapse	NA	NA
	Hypopharynx LPW	No collapse	Partial collapse	Complete collapse	NA	NA
	Tonsils	No collapse	Partial collapse	Complete collapse	NA	NA
	Tongue base	No collapse	Partial collapse with lingual tonsils	Partial collapse without lingual tonsils	Complete collapse with lingual tonsils	Complete collapse without lingual tonsils
	Epiglottis	No collapse	Partial collapse	Complete collapse	NA	NA
Koo, 2013 [35]	Level	Degree of obstruction	Configuration			
			Anteroposterior diameter	Lateral diameter	Contributing structure	
	Retropalatal	0 / 1 / 2	Palate +/−	LPW +/−	Tonsil +/−	
	Retrolingual		Tongue base +/−	LPW +/−	Epiglottis +/−	
Lee, 2015 [36]	Zone	Degree of obstruction				
	Soft palate	0, no obstruction				
	Lateral wall + tonsils	1, partial obstruction				
	Larynx + epiglottis	2, complete obstruction				
	Tongue base	Grade 1	More than >50% obstruction displacement compared to the supine awake state			
		Grade 2	More than >75% obstruction displacement compared to the supine awake state			
Herzog, 2015 [37]	Zone	Grade	Description			
	Pharyngeal collapse at velum level	Grade 1	No collapse			
		Grade 2	Lateral collapse <50% of lumen			
		Grade 3	Lateral collapse >50% of lumen			
		Grade 4	Circular collapse <50% of lumen			
		Grade 5	Circular collapse >50% of lumen			
	Uvula/palate contact to posterior pharyngeal wall	Grade 1	No contact			
		Grade 2	Contact of the tip of the uvula			
		Grade 3	Contact of the half of the uvula			
		Grade 4	Contact of the base of the uvula / palate			
	Dorsal movement of the tongue base	Grade 1	Valleculea completely visible			
		Grade 2	Valleculea partial visible			
		Grade 3	Valleculea not visible			
		Grade 4	Contact to the posterior pharyngeal wall			
	Pharyngeal collapse at tongue base level	Grade 1	No collapse			
		Grade 2	Lateral collapse <50% of lumen			
		Grade 3	Lateral collapse >50% of lumen			
		Grade 4	Circular collapse <50% of lumen			
		Grade 5	Circular collapse >50% of lumen			
Carrasco-Llatas, 2016 [14]	Structure	Degree of obstruction	Configuration			
	Soft palate (velum)	0: no obstruction 1: partial obstruction 3: complete obstruction	Anteroposterior	Lateral	Concentric	
	Oropharynx		Anteroposterior	Lateral	Concentric	
	Tongue base		X, not visualised	Lateral	X, not visualised	
	Epiglottis		Anteroposterior	Lateral	Concentric	
			Anteroposterior	Lateral	X, not visualised	
Veer, 2016 [38]	Zone	Grade	Description			
	Palate	P1	No flutter seen			
			Soft palate not obstructing the airway			
			Open airway between the oropharynx and nasopharynx			
		P2	Anteroposterior palatal obstruction Uvula dropping into the a collapsed airway inferiorly			
		P3	Circumferential palatal collapse			
	Tonsils	T1	No tonsillar involvement			
			Previous tonsillectomy			
		T2	Tonsils causing less than 50% obstruction using the anterior commissure as the horizontal midpoint			
		T3	Tonsils causing 50% or more obstruction of the airway			
	Lateral pharyngeal wall	L1	No lateral wall collapse			
		L2	Lateral pharyngeal wall collapse causing less than 50% obstruction of the airway using the anatomical and anterior commissure as the horizontal midpoint			
		L3	Lateral pharyngeal wall causing 50% or more obstruction of the airway			
	Tongue base	Tb1	Tongue base not affecting the airway			
			Tongue base not altering the normal resting position of the epiglottis (no posterior deflection seen)			
			Vallecula seen			
		Tb2	Less than 50% obstruction of the airway due to tongue base			
		Tb3	50% or more of the airway due to tongue base			
	Epiglottis	E1	No epiglottic trapdoor phenomenon			
		E2	Epiglottis collapses down upon the glottis aperture during inspiration			

### Drug-induced sedation classification system

#### Croft et al. 1991 [7]

Croft et al. proposed the first form of a DISE classification system which consists of three categories: Group A, Group B and Group C. Group A is simple palatal snoring. No obstructive event was observed. There was notable palatal vibration, occasional prolapse of the uvula into the nasopharynx and circumferential narrowing at the velopharyngeal level associated with the palatal vibration. Group B is single-level palatal obstruction. There was palatal obstruction at the velopharyngeal level. The obstructions were usually circumferential closure. Group C was multisegment obstruction. There were snoring and obstructive episodes simultaneously at the velopharyngeal and oropharyngeal level. The proposed classification system is easy to understand. However, it is not sufficient for detailed analysis and reporting of DISE findings.

#### Pringle et al. 1993 [24]

Pringle et al. proposed a new DISE classification system based on Croft et al. DISE classification system [7]. Grade 1 represents simple palatal snoring. There is no episode of upper airway obstruction; however, noisy snoring is observed. The noise is caused by the vibration of the soft palate and vibration of the walls of the velopharyngeal sphincter and upper oropharynx. The first observable pattern is the flapping of the soft palate during inspiration. The second observable pattern is a narrowing of the velopharyngeal area in an anteroposterior or circumferential configuration. Furthermore, the tonsil can be observed to bulge into the narrowing of the airway. Grade 2 represents single-level palatal obstruction. Upper airway obstruction occurs only at the velopharyngeal level. The severity of the obstruction can vary from mild to very severe obstruction. The configuration of the obstruction can either be anteroposterior or circumferential obstruction configuration. Grade 3 represents multisegmental obstruction with intermittent oro-hypopharyngeal collapse. Palatal level obstruction occurs with intermittent oro-hypopharyngeal involvement with each inspiratory effort. Grade 4 represents sustained multisegment obstruction. There is sustained multisegment obstruction throughout the obstructive episode. Grade 5 represents tongue base obstruction. The tongue base obstruction occurs either because the tongue base falls back onto the posterior pharyngeal wall or the posterior pharyngeal walls appearing to close down onto the tongue base. In addition, the tongue base could be forward but the epiglottis obscured the laryngeal inlet to obstruct the airway. Pringle and Croft grading system proposed a more detailed DISE classification system for assessing upper airway obstruction; however, it can be quite challenging to understand at an early onset especially for new DISE examiners.

#### Camilleri et al. 1995 [25]

Camilleri et al. proposed a simple grading system as a DISE classification system with three grading levels. Grade 1 represents palatal snorers. Grade 2 represents mixed snorers. Grade 3 represents non-palatal (tongue base). Uvulopalatopharyngoplasty (UPPP) was the recommended treatment for grade 1. Medical treatment combined with UPPP was recommended for grade 2. Medical treatment and other surgical treatment except UPPP were recommended for grade 3. Medical treatments include weight loss, mandibular advancement or continuous positive airway pressure. Other surgical treatments include nasal surgery, tonsillectomy laser to tongue base, maxillary or mandibular osteotomy, hyoid advancement and tracheotomy. The proposed classification system provided a framework for planning surgical treatment based on classified DISE. However, the proposed treatment model is not sufficiently robust.

#### Quinn et al.1995 [26]

Quinn et al. proposed a DISE classification system based on airflow dynamic, sound generation and obstruction at the palate, tonsils, epiglottis and tongue base. Palatal obstruction is characterised as massive reduction in cross-sectional area of the velopharynx during inspiration to either a funnel-shaped aperture or a transverse. Also, palatal obstruction was categorised as the narrowing of the velopharynx due to a circular aperture as the uvula swing back and forth. Tonsil obstruction was characterised as intermittent obstruction with vibration of the tonsils marked by medialization of the tonsils on inspiration with the formation of a narrow slit in the sagittal direction. Tongue base obstruction is characterised as the narrowing of the airway at the level of the tongue base with visible vibration of the tongue against the pharyngeal wall. Epiglottis obstruction was categorised an omega-shaped epiglottis obstruction or epiglottic obstruction due to the tip of the epiglottis falling back onto and vibrating against the posterior pharyngeal wall. This was the first study to relate upper airway obstruction in conjunction with upper airway obstruction classification based on DISE. Without a well-developed 3D airflow model, it is hard to appreciate the significance of DISE classification in conjunction with upper airway obstruction.

#### Sadaoka et al. 1996 [9]

Sadaoka et al. proposed a DISE classification system based on three groups (Group A, Group B and Group C) of sites of upper airway obstruction. Grade A represents single-level palatal obstruction. Palatal vibration and velopharynx obstruction are observed. Grade B represents a single-level tongue base obstruction. Only tongue base obstruction is observed. Grade C is multisegment obstruction. Circumferential closure from the velopharynx to the tongue base is observed. This DISE classification system provides a simplistic and easy to understand upper airway obstruction classification. However, it is limiting when a robust analysis is required.

#### Higami et al. 2002 [27]

Higami et al. proposed a DISE classification system based on different pharyngeal stenosis patterns during sleep. Pharyngeal stenosis patterns were classified into three categories: falling type, all-round type and bilateral type. The falling type is an obstruction type caused by the soft palate and tongue sinking due to gravity. All-round type is an obstruction type caused by submucosa of the pharynx. Bilateral type is an obstruction type where the palatine tonsils were hypertrophied. A simple treatment plan was noted based on the DISE classification and attempt to evaluate surgical treatment outcomes. However, long-term surgical outcomes were not explored.

#### Iwanaga et al. 2003 [28]

Iwanaga et al. proposed a DISE classification system to assess changes in airway morphology before and after surgery. The DISE classification system consists of five types of obstructions: soft palate obstruction, circumferential obstruction, tonsillar-type obstruction, root-of-tongue obstruction and mixed-type (palate and tongue) obstruction. Soft palatal type of obstruction was defined as an obstruction caused by a uvula and soft palate posteriorly in contact with the pharyngeal. Circumferential palatal obstruction was defined as full-circumference soft palatal obstruction involving the posterior and lateral pharyngeal walls during expiration. The tonsillar obstruction was defined as an obstruction where the palatine tonsils come in contact at the midline during expiration. Root-of-tongue obstruction was defined as a single-level obstruction at the back of the tongue only. Mixed obstruction was defined as an obstruction where the soft palate obstruction and the root of the tongue obstruct the airway simultaneously.

#### Kezirian et al. 2011 [29]

Kezirian et al. proposed VOTE classification for analysing and reporting DISE findings. VOTE classification is a DISE classification system for reporting DISE findings based on analysis of the severity and configuration of obstruction at four anatomical levels: velum, oropharynx, tongue base and epiglottis. The severity of obstruction can be classified as 0, no obstruction (no vibration, < 50%); 1, partial obstruction (vibration 50–75%) and 2, complete obstruction (collapse, > 75%).

The configuration of obstruction at the velum level can be in anteroposterior, lateral or concentric direction. Oropharynx obstruction occurs in the lateral and concentric direction. Tongue base obstruction occurs only in the anteroposterior configuration. The configuration of epiglottis obstruction occurs in the anteroposterior direction. VOTE classification provides a clear and simple framework for robustly and accurately analysing upper airway obstruction. However, VOTE can be perceived to have oversimplified upper airway obstruction.

#### Vicini et al. 2012 [30]

Vicini et al. proposed the nose oropharynx hypopharynx and larynx (NOHL) as a DISE classification system for diagnostic standardised examination for OSA patients. NOHL classification system is focussed on assessing the severity and configuration of upper airway obstruction based on four anatomical zones: nose, oropharynx, hypopharynx and larynx. The severity of obstruction was categorised in conjunction with the Müller manoeuvre grading system. Grade 1 is an upper airway obstruction between 0 and 25% airway collapse and also defined as no collapse during Müller manoeuvre. Grade 2 is an upper airway obstruction between 25 and 75% airway collapse. Grade 3 is an upper airway obstruction between 50 and 75% airway collapse. Grade 4 is an upper airway obstruction greater than 75% airway collapse and also defined as a total pharyngeal wall collapse during Müller manoeuvre. A generalist approach of anteroposterior, lateral and concentric configuration of obstruction was applied to the nose, oropharynx and hypopharynx. The laryngeal obstruction was assessed as positive or negative at the supraglottic and glottic level. The key advantage of NOHL classification is the provision of a scoring framework for upper airway obstruction during awake and sleep state. Furthermore, NOHL classification can potentially aid therapeutic decision-making and surgical outcome analysis. The key limitation is that two of these anatomical zones (nose and larynx) play significantly reduced roles in upper airway obstruction. [31] Also, there is no sufficient evidence on the potential of NOHL classification aiding therapeutic decision-making and surgical outcome analysis.

#### Bachar et al. 2012 [32]

Bachar et al. 2012 proposed endoscopic grading system and severity index for accurately understanding upper airway obstruction patterns. The endoscopic upper-airway grading system is a DISE classification system that comprises of five anatomical obstruction sites: nose and nasopharynx (N), palatine plane or tonsils (P), tongue base (T), larynx (L) and hypopharynx (H). All forms of obstruction are categorised as either partial (1) or complete (2). It is important to note that anatomic level without obstruction is not labelled. The endoscopic upper-airway grading system uses a severity index (SI). The endoscopic sleep index is calculated by adding the digits as representation of the obstruction pattern at each level. The key novelty of the endoscopic upper-airway grading system is the concept of a formulaic calculated cumulative severity of obstruction for precision classification. However, severity index does not account for the configuration of an upper airway obstruction.

#### Victores et al. 2012 [33]

Victores et al. proposed upper airway obstruction classification system assessing surgical outcomes on OSA patients called upper airway obstruction classification system. The upper airway obstruction classification system consists of four anatomic zones: palate (velum), lateral wall/tonsillars, tongue base and epiglottis [29]. The degrees of obstruction for all anatomic zones are partial (≤75% obstruction) and complete (≥75%). The sustainability of obstruction for tongue base and epiglottis is intermittent and sustained obstruction [24]. Intermittent obstruction is an obstruction only during inspiration in an apnoeic episode. Sustained obstruction is an obstruction throughout an apneic episode. Intermittent and sustained obstructions of the palate and lateral wall/tonsillar pillars were not visualised. This was the first study to combine degree of obstruction and sustainability of obstruction. Its key limitation was classification based on only nasal surgery effect.

#### Gillespie et al. 2012 [34]

Gillespie et al. proposed DISE index as DISE classification to provide a clear framework for clinical airway function analysis, assisting surgical planning and standardised DISE techniques, training and interpretation. DISE index classifies the degree of anatomic collapse of the upper airway on an ordinal scale from 0 (no collapse) to 12 (multilevel, complete collapse).The anatomical zones examined are as follows: palate, lateral walls of hypopharynx, tonsils, tongue base and epiglottis. Obstruction at the palate, lateral walls of hypopharynx, tonsils and epiglottis can be categorised into no collapse, partial collapse and complete collapse. Obstruction at the tongue base is categorised as no collapse, partial collapse with lingual tonsils, partial collapse without lingual tonsils, complete collapse without lingual tonsils and complete collapse with lingual tonsils. The key contribution of the DISE index is showing a relationship between classification and treatment planning. However, the relationship between classification and treatment plan is not well defined.

#### Koo et al. 2013 [35]

Koo et al. proposed a different DISE classification system based on modifying the VOTE classification. The modified VOTE classification system is based on two anatomic levels: retropalatal level (the region of posterior to the soft palate) and retrolingual level (the region of the pharynx posterior to the vertical portion of the tongue). The retropalatal level was subdivided into the palate (anteroposterior diameter), lateral pharyngeal wall (lateral diameter) and tonsil (specific structure contributing to obstruction). The retrolingual level consists of the tongue base (anteroposterior diameter), lateral pharyngeal wall (lateral diameter) and epiglottis (specific structure contributing to obstruction). The severity of obstruction was categorised as no obstruction (0), partial obstruction (1, 50–75%) and complete obstruction (2, >75%). The key contribution of the proposed classification system is the attempt to relate upper airway obstruction to BMI and AHI. Further, a population-driven classification system is proposed for the first time (Korean population). Its key limitation small population size and scope.

#### Lee et al. 2015 [36]

Lee et al. proposed a DISE classification system to evaluate upper airway obstruction based on sleep position. This study proposed a DISE classification based on four anatomic zones: soft palate, lateral wall including palatine tonsils, tongue base and larynx including epiglottis. The degree of obstruction is as follows: 0, no obstruction; 1, partial obstruction (vibration with desaturation) and 2, complete obstruction (total collapse of airway with desaturation). Tongue-based obstruction was categorised as grade 1 (more than 50% obstruction displacement compared to the supine state) and grade 2 (more than 75% obstruction displacement compared to the supine state). This was the first study to propose a classification system combining upper airway obstruction and sleep position. However, the characterisation of upper airway obstruction and sleep position was not well defined.

#### Herzog et al. 2015 [37]

Herzog et al. proposed a DISE classification system based on the vibration and collapsibility of the upper airway. Four vibration and collapsibility levels of the upper airway were examined: pharyngeal collapse at velum level, uvula/palate contact to posterior pharyngeal wall, dorsal movement of the tongue base and pharyngeal collapse base level. Pharyngeal collapse at velum level was graded 1–5 based on lateral or circular pattern of collapsibility. The contact of the uvula/soft palate to the posterior pharyngeal wall is classified as grades 1–4 based on anteroposterior pattern of vibration. The dorsal movement of the tongue base is classified as grades 1–4 depending on the visibility of the valleculae and contact to the posterior pharyngeal wall based on an anteroposterior pattern of movement. The pharyngeal collapse of the tongue base level is graded 1–5 depending on a lateral and circular pattern of collapsibility. The key novelty of this DISE classification system is having well-defined classification boundaries and relating vibration and collapsibility of the upper airway obstruction during simulated sleep and DISE. The key limitation of this classification system that simulated snoring is significantly not representative on sleep.

#### Carrasco-Llatas et al. 2016 [14]

Carrasco-Llatas et al. proposed another modified VOTE classification system very similar to the original VOTE classification. The proposed modification is lateral and concentric configuration of tongue base obstruction which is not considered in the original VOTE classification.

#### Veer 2016 [38]

Veer proposed P-T-L-Tb-E classification system as DISE classification system that is simple to learn, objective and easy to remember. P-T-L-Tb-E classification system is characterised by severity and configuration on upper airway obstruction based on five anatomical structures: palate (P1, P2, P3), tonsils (T1, T2, T3), lateral pharyngeal wall (L1, L2, L3), tongue base (Tb1, Tb2, Tb3) and epiglottis (E1, E2). P1 grade represents no fluttering of the palate, soft palate not obstructing and open airway between the oropharynx and nasopharynx. P2 grade represents a palatal anteroposterior obstruction and/or uvula obstruction due to dropping into a collapsed airway inferiorly. P3 grade represents a palatal circumferential collapse. This is often seen with lateral wall collapse inferiorly. T1 grade represents no tonsil obstruction. T2 grade represents less than 50% tonsil obstruction of the airway using the anterior commissure as the horizontal midpoint. T3 grade represents greater than 50% tonsils obstruction of the airway. L1 grade represents no lateral wall obstruction. L2 grade represents less than 50% lateral pharyngeal wall obstruction of the airway using the anatomical midline and the anterior commissure as the horizontal midpoint. L3 grade represents greater than 50% lateral pharyngeal wall obstruction of the airway. Tb1 grade represents no tongue base obstruction, tongue base not altering the normal resting position of the epiglottis and vallecula seen. Tb2 grade represents less than 50% tongue base obstruction where the airway is defined as the space between the anterior commissure and the pharyngeal wall. Tb3 grade represents more than 50% tongue base obstruction. E1 grade represents no epiglottic trapdoor phenomenon. E2 grade represents epiglottis trapdoor phenomenon. P-T-L-Tb-E classification system provides a robust classification system, and scoring framework for analysis DISE results with well-defined boundary conditions. Furthermore, P-T-L-Tb-E classification system provides a well-defined classification framework for computational analysis. The visualisation of the anterior commissure during DISE might be quite challenging.

## Discussion

Our systematic review identified 17 studies that proposed 14 different new systems and 3 modified DISE classification systems for analysing anatomic findings based on drug-induced sleep endoscopy. This systematic review identified five findings.

First, there is currently no universally accepted DISE classification system for analysing anatomic levels/structure and severity of obstruction. Some classification systems preferred to use anatomical levels while others preferred anatomical structures (Table 2). Furthermore, some classification systems preferred to use three degrees of severity—none, partial and complete obstruction while others used a semi-quantitative system with 0–25%, 25–50%, 50–75% and 75–100% of obstruction (Table 2). There seems to be an agreement on three configurations of obstruction especially within the European community. Hence, the null hypothesis of a universally accepted DISE classification system for analysing DISE findings is nullified. The alternative hypothesis that there are many DISE classification systems for analysing DISE findings is true. Furthermore, it was observed that there is a growing number of DISE classification systems. This further makes the notion of having one universally accepted DISE classification system harder to achieve. It is important to note that there are no studies demonstrating how clinical findings can be mapped from one DISE classification system to another. Furthermore, the lack of open dataset impedes developing a relational map between different DISE classification systems.

Second, it was observed that a majority of DISE-related studies identified in our broader literature search captured, analysed and reported only anatomical information during DISE (Table 2). These studies demonstrated that DISE was an effective tool for assessing anatomical features of OSA. It was observed that cardio-respiratory parameters (blood oxygen saturation, electrocardiogram and blood pressure) from a standard anaesthesiological monitoring system (or a medical sensor) and depth of sedation using bispectral monitoring system was not recorded. Arguably, one can assert that an identified anatomical obstruction in a DISE video does not lead to a significant drop in saturated blood oxygen level. Furthermore, one can argue that an observed obstruction in a DISE video was due to sedation effect rather than a natural cause. Hence, anatomical information from DISE video is insufficient for objectively describing an obstructive event during DISE. Therefore, it is necessary to capture multiple information which consist of at least DISE video data, cardio-respiratory parameters and bispectral index is required for objectively classifying DISE findings [39].

Third, it was observed that there is no robust DISE classification treatment planning framework for deciding appropriate surgical treatment for OSA. Some studies proposed different DISE classification treatment planning frameworks (Table. 2). However, there was insufficiently level of evidence to justify the proposed framework. As noticed earlier, the growing number of DISE classification system poses multiple ways of devising a surgical treatment plan. This makes the process of deciding a surgical treatment for OSA biased toward choice of classification system and personal experience. Furthermore, there is no randomised trial evaluating different DISE classification treatment planning frameworks for OSA management.

Fourth, it was observed that there is no robust DISE classification treatment outcome framework for predicting the likelihood of surgical treatment for OSA management. Some studies proposed showing a before and after surgical result based on a DISE classification system (Table. 2). This raises fundamental questions on the process of how DISE classification affects the outcomes of performed surgical operations. Does performed surgical procedure lead to higher likelihood of better sleep in an OSA patients? Are suboptimal surgeries performed? Given that there is no randomised clinical trial, it remains an unanswered question.

Fifth, all DISE classification required a human examiner (Table. 2). The level of experience of a human examiner plays a key role in the accuracy of the DISE assessment: junior ENT surgeons are prone to more errors [21]. This creates a case for a computational assistive tool for DISE classification with the aim of nullifying experience bias and increased classification objectivity.

Lastly, additional research is needed [31]. Further studies to develop one universally accepted classification system is critically needed. A prospective study mapping different DISE classification systems, and its classification results will be helpful in developing a framework for relating different DISE findings from different studies and different research and clinical centres. Coming up with a validated framework for relating DISE classification results to surgical treatment plan and surgical treatment outcome will provide an adequate basis for the surgical treatment decision. Further research is required to understand the minimum required dataset to objectively and accurately describe DISE findings. Research into computational DISE classification of DISE findings will provide an alternative method to human-dependent classification process. Finally, there needs to be an open DISE dataset for a more multidisciplinary multicentred research in DISE.

### Statement of significance

First, there is a growing number of DISE classification systems which increase subjectively in the analysis of upper airway obstruction using DISE making DISE findings incompatible across different studies. Second, there is no standardised framework for deciding, planning and predicting surgical outcome based on any current DISE classification system which increases the likelihood of suboptimal surgical treatment decision, planning and outcomes for OSA management. Third, given that human analysis is required for analysing anatomical findings in DISE videos, there is a growing divergence in analytic analysis between different clinical and research centres. Hence, a computational approach would be beneficial to serve as a secondary classifier.

### Limitations

This systematic review is limited by the lack of published studies on DISE classification systems based randomised control trials. Although authors have exhaustively and systematically searched the literature, there is always a possibility of not finding published studies on new and modified DISE classification systems. Furthermore, our systematic reviews primarily focused on DISE classification system at its development stage which limits our report on the evolution of each DISE classification system examined.

## Conclusions

The methodology of classifying DISE findings is important in determining the right clinical findings on upper airway obstruction and patient selection. There is currently no universally accepted DISE classification system. Furthermore, there is currently no universally accepted framework for devising a treatment plan and establishing the likelihood of surgical outcome based on any DISE classification systems. VOTE classification system is currently the most adopted and widely used DISE classification system. Two adequate alternative to VOTE classification system are NOHL classification system or P-T-L-Tb-E classification system based on its simplicity and its ease of remembrance. Additional multidisciplinary multicentred research is required to develop a universally objective DISE classification system.
